# Proteome-wide AlphaFold pool party

**DOI:** 10.1038/s44320-026-00198-6

**Published:** 2026-02-17

**Authors:** Kevin Drew

**Affiliations:** Department of Biological Sciences, University of Illinois at Chicago, Chicago, IL 60607 USA

**Keywords:** Computational Biology

## Abstract

K. Drew highlights a recent study by Todor and colleagues (in this issue of *Molecular Systems Biology*) that presents an innovative strategy to systematically map all protein interactions in *M. genitalium* using AlphaFold3.

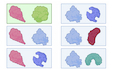

## Teaser

An outstanding challenge in molecular biology is the production of a complete and accurate set of protein interactions for a given organism. Todor et al, introduce a clever strategy to efficiently screen all protein interactions in *Mycoplasma genitalium* using AlphaFold3 (Todor et al, 2026).

**Figure 1:**
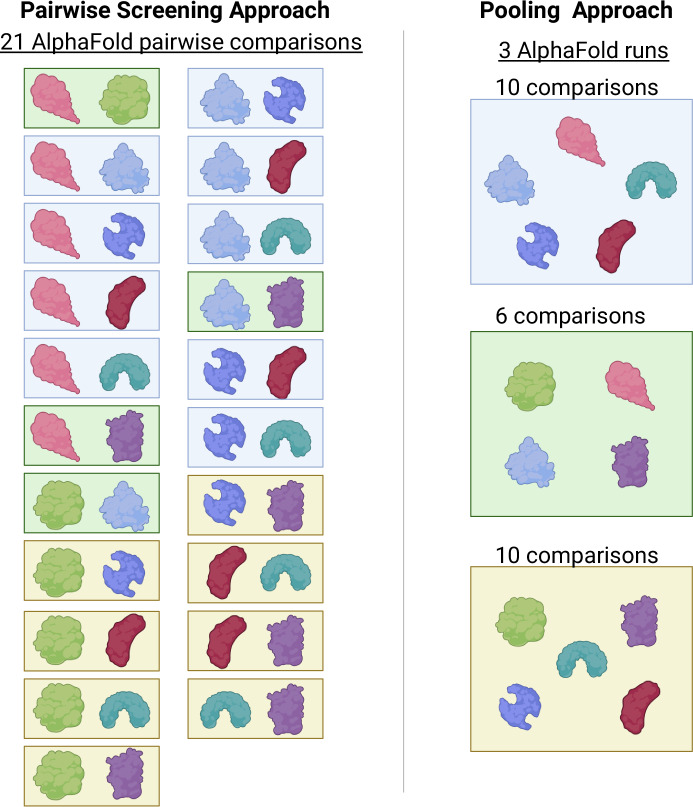
Proteome-wide pooled protein interaction screening using AlphaFold3. Todor et al developed a strategy to efficiently screen protein interactions for an entire proteome. AlphaFold can model protein interactions of two proteins that have not previously been known to interact. Groups have therefore utilized AlphaFold to screen hundreds of thousands of potential pairwise interactions. The traditional approach runs AlphaFold for each pair of proteins, resulting in hundreds of thousands to hundreds of millions of runs, a computationally intractable task. The left panel shows the enumerated pairwise interactions for a set of seven proteins resulting in 21 AlphaFold runs. Todor et al,’s strategy runs AlphaFold on random pools of proteins. The right panel shows the pooling approach on the same seven proteins, resulting in only 3 AlphaFold runs. Overall, the pooling approach is deemed more computationally efficient as well as more accurate. Image made with BioRender.

Recent advances in the field of computational structure prediction allow researchers to computationally screen possible protein pairs for their propensity to interact. While impressive, screening all pairs in an organism is still largely computationally intractable due to limited resources. Todor et al approached this challenge from a new angle. Instead of running all possible pairs individually, they rationalized that they could evaluate groups of proteins together (Fig. 1). Since few interactions are relevant out of the vast total number of possible pairs, additional proteins are unlikely to interfere with the evaluation of true interactions. Therefore, they selected random sets of proteins or “pools”, generally containing between 10 and 25 proteins. They then ran all proteins in a pool through AlphaFold3’s free online server simultaneously. This produced all vs all comparisons within the pool. Moreover, the random pools were constructed so every pair of proteins is present in a pool at least once; therefore, all pairs of proteins will be modeled when all pools are run through AlphaFold3. Compared to the pairwise approach, the pooled approach reduced the total number of jobs 100-fold, improved the runtime by twofold, and somewhat unexpectedly, improved the overall accuracy of identifying true interactions. So not only was the approach more efficient, but more accurate too.

The organism selected for study by Todor et al, *Mycoplasma genitalium*, has a reduced genome containing ~475 proteins, resulting in 113,050 possible pairwise interactions. As the total possible pairwise interactions scale quadratically with the size of the proteome, *Mycoplasma genitalium*’s possible pairwise interactions is considerably less than the ~200 million possible pairwise interactions in the human proteome. Therefore, the strategy alone is insufficient to solve the problem outright for all organisms. Other groups have approached this scaling problem by prioritizing pairs of proteins most likely to interact by other evidence (Humphreys et al, 2021; Burke et al, 2023; Jänes et al, 2024; Zhang et al, 2024; Gao et al, 2022; Schmid et al, 2025). For example, one of the first efforts applied to human, Burke et al produced AlphaFold2 models of confident protein pairs found in publicly available protein interaction databases (hu.MAP2.0 (Drew et al, 2021), HuRI (Luck et al, 2020)). Prioritization based on experimental data does require the data to exist in the species of study (or related species). Non-model organisms often lack these data, and so the pooling approach is a great addition to the computational toolkit for understudied organisms. In addition, there is nothing conflicting between the approaches of pooling and prioritization (when data is available). This allows the potential for them to be used in conjunction if combined in future efforts.

This work is a significant and productive advance, but additional challenges remain. Understanding the underlying nature of why the pooled approach is more accurate than the pairwise approach is currently unclear. The authors make several hypotheses, including limiting false positives by increasing competition among interactions. Since the pooling approach evaluates many protein interactions at the same time, only the most confident interaction at a specific protein interface will be selected. This would eliminate false positives, especially for promiscuous proteins. A better understanding of the increase in accuracy will allow for the engineering of protein pools that optimize accuracy.

The hypothesis above, however, uncovers a limitation in the pooling approach. If AlphaFold3 is prioritizing some interactions based on spatial constraints, protein interactions that are mutually exclusive, that is, two proteins that bind a third protein at the same interface, one protein may be prioritized over the other. Many mutually exclusive interactions exist in nature and often alter molecular functions in different cellular contexts. The degree of this issue depends on the number of mutually exclusive interactions and the size of the pools. With the current setup, this is likely to be rare, but if completeness is the goal (or pool size is increased), it will need to be addressed.

Since the Todor et al method relies on AlphaFold3, it is important to consider the limitations of AlphaFold itself. AlphaFold uses a protein’s multiple sequence alignment to identify co-evolving pairs of amino acids within a protein or between proteins for protein interactions. The algorithm uses the co-evolving amino acids as distance constraints when building a structural model. Proteins without sufficiently deep multiple sequence alignments have been shown to suffer greatly in accuracy. Around 40 proteins in *Mycoplasma genitalium* (~8%) have limited sequence homology, likely only found in this species. This results in nearly 18,000 protein pairs that cannot be evaluated by this approach due to insufficient multiple sequence alignments. These potential interactions may be responsible for the unique biology of *Mycoplasma genitalium*, not conserved in other organisms. Context-specific interactions are similarly difficult to discern, such as interactions that are only present under certain cellular conditions or cell types.

Finally, pool-wise interaction screening has the ability to identify higher-order interactions such as trimers, tetramers, etc., which are more biologically relevant than pairwise screening. Unfortunately, generating purely random pools limits the probability of having three or more proteins that interact in the cell in the same pool. Using a pooled strategy to capture all pairwise interactions is likely to be effective, but randomly screening all possible three-way ( ~ 18 million combinations in *Mycoplasma genitalium*) interactions will likely be out of reach for quite a while without intelligently choosing pools.

In closing, Todor et al's effort brings us closer to scaling all vs all computational screening of protein interactions for model and non-model organisms. The efficiency and boost in accuracy suggest an indispensable tool to reach for in our rapidly increasing toolkit.
